# Impact of missing data on bias and precision when estimating change in patient-reported outcomes from a clinical registry

**DOI:** 10.1186/s12955-019-1181-2

**Published:** 2019-06-20

**Authors:** Olawale F. Ayilara, Lisa Zhang, Tolulope T. Sajobi, Richard Sawatzky, Eric Bohm, Lisa M. Lix

**Affiliations:** 1 Department of Community Health Sciences, University of Manitoba, S113-750 Bannatyne Avenue, Winnipeg, MB R3E 0W3 Canada; 2 Department of Community Health Sciences Cumming School of Medicine, University of Calgary, Calgary, AB Canada; 3 School of Nursing, Trinity Western University, Langley, BC Canada; 4 Department of Surgery, University of Manitoba, Winnipeg, MB Canada

**Keywords:** Auxiliary variables, Maximum likelihood estimation, Missing data, Registry, Mixed-effects model

## Abstract

**Background:**

Clinical registries, which capture information about the health and healthcare use of patients with a health condition or treatment, often contain patient-reported outcomes (PROs) that provide insights about the patient’s perspectives on their health. Missing data can affect the value of PRO data for healthcare decision-making. We compared the precision and bias of several missing data methods when estimating longitudinal change in PRO scores.

**Methods:**

This research conducted analyses of clinical registry data and simulated data. Registry data were from a population-based regional joint replacement registry for Manitoba, Canada; the study cohort consisted of 5631 patients having total knee arthroplasty between 2009 and 2015. PROs were measured using the 12-item Short Form Survey, version 2 (SF-12v2) at pre- and post-operative occasions. The simulation cohort was a subset of 3000 patients from the study cohort with complete PRO information at both pre- and post-operative occasions. Linear mixed-effects models based on complete case analysis (CCA), maximum likelihood (ML) and multiple imputation (MI) without and with an auxiliary variable (MI-Aux) were used to estimate longitudinal change in PRO scores. In the simulated data, bias, root mean squared error (RMSE), and 95% confidence interval (CI) coverage and width were estimated under varying amounts and types of missing data.

**Results:**

Three thousand two hundred thirty (57.4%) patients in the study cohort had complete data on the SF-12v2 at both occasions. In this cohort, mixed-effects models based on CCA resulted in substantially wider 95% CIs than models based on ML and MI methods. The latter two methods produced similar estimates and 95% CI widths. In the simulation cohort, when 50% of the data were missing, the MI-Aux method, in which a single hypothetical auxiliary variable was strongly correlated (i.e., 0.8) with the outcome, reduced the 95% CI width by up to 14% and bias and RMSE by up to 50 and 45%, respectively, when compared with the MI method.

**Conclusions:**

Missing data can substantially affect the precision of estimated change in PRO scores from clinical registry data. Inclusion of auxiliary information in MI models can increase precision and reduce bias, but identifying the optimal auxiliary variable(s) may be challenging.

## Background

Clinical registries are databases that capture information about the health and healthcare use of patients having a specific health condition or healthcare treatment. Patient-reported outcomes (PROs) are increasingly collected in clinical registries because they provide valuable information about the patient’s perspectives on their health, including pain, perceived functional abilities, and mental health [1]. PRO data in registries can be a useful tool for clinicians to assess quality of care and improvements in patient health status, beyond what can be captured from objective measures of health status such as complication rates and patient mortality [2]. Clinical registry data have a number of other potential uses, including evaluations of new programs and treatments. Registry data are also used for research. However, clinical registry data collection and evaluation may not always follow the same methods or practices as are used in research studies involving primary data collection [3]. Clinics may also not have the resources needed to routinely and thoroughly check the data for accuracy and completeness.

Studies involving clinical registry data are often longitudinal in nature; for example, they may examine change in PROs before and after an intervention or healthcare treatment [3]. Longitudinal study findings may be strongly influenced by missing data, which can arise when participants die, miss scheduled clinic visits, or fail to respond to clinic questionnaires or interviews. One potential consequence of missing data in a longitudinal study is a loss of power to detect change. Missing data can also result in under- or over-estimation of treatment effects, depending on its characteristics [3–5].

The choice of methods to handle missing data is generally dependent on the missingness mechanism [6–8]. According to Little and Rubin’s taxonomy, these mechanisms can be categorized as missing completely at random (MCAR), missing at random (MAR), or missing not at random (MNAR) [8]. Data are MCAR if the reason for the missingness is unrelated to the outcomes. MAR arises if the reason for dropout depends on the observed outcomes and possibly on observed covariates at any or all occasions before the individual is lost to follow up. The MNAR mechanism depends, in whole or in part, on unobserved measurements.

In longitudinal studies, commonly used missing data methods include list-wise deletion, complete-case analysis (CCA), average available observation carried forward, last observation carried forward, and conditional or unconditional mean imputation [9–11]. However, these methods may result in a loss of statistical power and biased estimates of change, especially when data are MNAR. Other missing data methods, including maximum likelihood (ML) and multiple imputation (MI), which are practical to implement in real-world data and increasingly being adopted, are recommended when the missing data mechanism is ignorable, that is, when the distribution of the missing data indicator is independent of the missing data, conditional on the observed data [11]. Beyond these methods, machine-learning algorithms such as the k-nearest neighbor method, decision trees, and random forest imputation, which are used to construct predictive models to estimate observations that will replace the missing values, can be used when the missing data mechanism is ignorable [12]. However, these machine-learning algorithms may distort the data distribution or introduce spurious associations when not carefully implemented to address missing data [13].

Other types of registries have used ML and MI methods to address missing data. For example, in cancer registries, MI methods based on specific clinical features have been used to impute missing prostate cancer stage information [14]. In a trauma registry, O’Reilly et al. (2012) identified and handled incomplete data using the MI method [15]. In a national weight control registry, Thomas et al. (2014) addressed missing data using the ML method in their evaluation of the effect of behavior change on weight-loss trajectories [16]. Similarly, in obesity surgery and medical birth registries, missing observations on the outcome variables were addressed using the ML method [17, 18]. However, for all of the studies, the use of these methods is predicated on the assumption that the data are MAR.

When data are MNAR or the missingness mechanism is non-ignorable, methods such as pattern mixture models, shared parameter models, and selection models are recommended [3, 19]. However, these methods are less frequently used because the missing data mechanism must be modeled and they can be computationally intensive [11].

Another approach to ensure that the assumption about ignorability of the missing data is plausible is to use auxiliary (i.e., supplementary) variables that are potential correlates of missingness and/or the outcome of interest [20]. The use of auxiliary variables related to the outcome of interest may reduce the bias due to missing data in model estimates by adding information associated with missingness to the model. Auxiliary variables are typically found in external data sources. An example of a data source that may contain useful auxiliary variables is administrative health data, which captures information about healthcare use and health status of patients; the advantage of administrative data is that they are routinely collected for purposes of health system management or remuneration, so they are unlikely to have missing values. Auxiliary variables are generally not of direct interest, other than for keeping the assumptions about ignorability of the missing data plausible [20, 21].

Several studies have shown that the theoretical advantage of auxiliary variables is the same for ML and MI methods [3, 20]. However, it is more straightforward to include auxiliary variables without adjusting the analysis model in MI,when compared to including them in the ML model [21, 22]. Previous studies have compared MI with other missing data methods using simulated data drawn from a real-world cohort, to preserve the complex relationships amongst the covariates and the outcomes in a longitudinal setting [6, 23]. However, neither the precision nor the bias of MI with and without auxiliary variables in PROs from clinical registry have been previously compared.

The primary purpose of this study was to compare the impact of several missing data methods on the precision of the estimated change in PRO measures in longitudinal data from a clinical registry. A secondary purpose was to use computer simulation to demonstrate the potential effects of including an auxiliary variable in the imputation model on bias and precision of PRO change estimates in longitudinal data.

## Methods

### Data source and cohorts

Study data were from the population-based Winnipeg Regional Health Authority (WRHA) Joint Replacement Registry. The WRHA is the largest health region in the central Canadian province of Manitoba, which has a population of approximately 1.2 million residents. The Registry captures more than 90% of all hip and knee replacement surgeries performed within the health region and approximately 75% of all replacement surgeries conducted in the province.

Information contained in the Registry includes age, sex, medical conditions, implant details, complications, and both general and condition-specific PROs. These data are collected via self-report and chart abstraction from medical records [24, 25]. Much of the information is collected at a pre-operative assessment conducted approximately one month prior to surgery, with additional data collection at one year following surgery.

The study cohort included all patients in the Registry who had total knee arthroplasty (TKA) between April 1, 2009 and March 31, 2015. Patients with inaccurate data on sex and BMI were excluded. The simulation cohort was comprised of patients from the study cohort who had complete information on PRO measures, demographic, and health status measures.

### Study measures

Generic and condition-specific PROs in the WRHA Joint Replacement Registry are collected via self-report questionnaires completed in the pre-operative assessment clinic and via mailed self-report questionnaires completed one year following surgery. We limited our attention in this study to the generic Short Form Survey version 2 (SF-12v2), a 12-item generic measure of physical and mental well-being. It produces Physical Component Summary (PCS) and Mental Component Summary (MCS) scores, which can range in value from 0 (worst) to 100 (best). Scores are normalized so that values above or below 50 are better or worse, respectively, than their corresponding values in the general population [26].

Demographic information on patient age and sex are extracted from patients’ medical records and included in the Joint Replacement Registry. Age was defined at the time of the pre-operative assessment. Body mass index (BMI) was calculated from self-reported weight and height at the pre-operative assessment. Information about comorbid health conditions, such as heart disease, were also obtained via self-report at the pre-operative assessment.

### Missing data methods

ML and MI methods were selected for use in this study because of their efficient computational requirements and recommendations in the literature for their adoption in practice [11]. The ML method chooses parameter values that assign the maximum possible probability or probability density to the observed data under a well-defined family of parametric probability models. The probability or probability density of the realized data is the likelihood function [11]. The missing values are removed from the likelihood by a process of summation or integration. These likelihood functions have a complicated form that requires special computational techniques such as expectation maximization [27]. Estimates obtained using this method are unbiased if the missing data are MAR and the statistical model has been correctly specified.

For the MI method, each missing value is replaced by *M* > 1 imputed values. Each value is a Bayesian draw from the conditional distribution of the missing observation given the observed data. The imputations are expected to represent the information about the missing values that is contained in the observed data for the chosen imputation model. MI involves three distinct tasks: (a) missing values are filled in *M* times to generate *M* complete data sets, (b) the complete data sets are analyzed using standard procedures, and (c) results from the *M* analyses are combined into a single inference estimate. The efficiency of the estimate is dependent on the number of imputations and fraction of missing information [28, 29]. Similar to the ML approach, the MI procedure also relies on the assumption that data are MAR. However, the process of handling missing data differs. In MI, missing values are treated in a step that is completely separate from the analysis. This separation has both positive and negative consequences. On the negative side, there is the possibility that a researcher may proceed to analyze the imputed data without considering how the imputations were generated. For example, a model based on a multivariate normal distribution allows pairwise associations among variables but not interactions. Therefore, imputed data set may tend to exhibit interactions that are weaker than those found in the population. On the positive side, the model is flexible and a straightforward process to include auxiliary variables. It is also not necessary to include these auxiliary variables in subsequent analyses, as their full effect is taken into account during the MI process, and is automatically carried forward into subsequent analyses of the imputed data [20, 30].

### Statistical analysis

Descriptive statistics including means, standard deviations (SD), frequencies, and percentages were used to describe the cohorts at the baseline (i.e., pre-operative) measurement occasion. Patterns of missing data were described for the study cohort using percentages.

A linear mixed-effects model was used to estimate change in SF-12v2 PCS and MCS scores between pre- and post-operative occasions; the choice of models and covariates was based on previous research with these data [31]. Specifically, the model included a random intercept and multiple fixed covariates, including time, age, sex (male [reference], female), and body mass index (BMI < 24.9, 25.0-29.9, 30.0+ [reference], comorbid chronic conditions (including heart disease, depression, high blood pressure, diabetes and back pain (No [reference], Yes)). The two-way interaction of sex and time was included in the model based on preliminary assessments of model fit using penalized likelihood-based fit statistics (e.g., Akaike Information Criterion).

Mixed-effects regression models based on CCA, ML, and MI methods were applied to the study cohort data; separate analyses were conducted for the PCS and MCS. CCA was conducted for the subset of patients who had no missing observations on any variables at either the pre- or post-operative occasions. For the MI method, Markov Chain Monte Carlo (MCMC) sampling of the full predictive distribution was adopted; it assumes a multivariate normal distribution for the imputations. This assumption was descriptively assessed using quantile-quantile plots of the observed values. Ten imputations were conducted, as this number has been shown to be sufficient for achieving a reasonable efficiency for high proportions of missing observations [29].

All analysis were carried out in R using the lme function [32] and multiple imputation by chained equations [33].

### Simulation study

The simulation study was conducted next. In our analysis of the simulation cohort data, we used all variables previously described for the study cohort in addition to a single hypothetical auxiliary variable, *Z,* which was generated from a bivariate normal distribution. Specifically, *Z* was correlated with both the pre- (*Y*_*1*_) and post-operative (*Y*_*2*_) scores with *ρ* = corr (**Y**, *Z*) = 0.2, 0.5 and 0.8, where **Y** = (*Y*_1_, *Y*_2_).

Random samples of size *n* = 1000 were selected from the simulation cohort; mixed-effects models, as specified previously, were applied to PCS scores. Pre-specified amounts (10%, 25% and 50%) of data were removed from the outcome variable via MCAR, MAR, and MNAR mechanisms by modeling the probability of the missing indicator conditional on the outcome variable using a logistic regression model. The ML, MI and MI-Aux (i.e., multiple imputation with *Z* included the imputation model) methods were used to address missingness.

A total of 1000 replications were conducted for each of the 27 simulation conditions, which were obtained by crossing all possible combinations of types and amounts of missingness with the magnitude of correlation of the hypothetical auxiliary variable with the outcome measure. We evaluated bias and error in the regression parameter estimates including the intercept (*β*_0_), which is the estimated average PRO score at the pre-operative occasion, change (*β*_*T*_) between the pre- and post-operative occasions, and time-sex interaction (*β*_*TS*_). Specifically, we computed standardized bias, root mean squared error (RMSE), 95% confidence interval (CI) coverage, and the average width of the 95% CI for each regression parameter mentioned above. Standardized bias was the ratio of the bias, the difference between the estimates obtained from the model applied to the random sample with n = 1000 obervations, and all data in the simulation cohort, and the SD of the estimates expressed as a percent; smaller values indicate less bias. The RMSE was calculated from the sum of squared bias and variance; smaller values indicate less error. Coverage was calculated as the proportion of the replications for which the 95% CI contained the true value of the parameter of interest; good performance is evident when the actual coverage is approximately equal to the nominal coverage rate of 95%. The average width of the 95% CI was the difference between the upper and lower limits of the interval averaged over the number of replications. Shorter intervals imply greater precision and higher power, provided the 95% CI coverage is high.

## Results

### Description of cohorts and missing data

Table 1 describes characteristics of the study and simulation cohorts. The average age of the TKA patients was approximately 67 years in both cohorts. More than half of the patients were obese. The most common chronic conditions were high blood pressure and back pain.

**Table 1:** Pre-operative Characteristics of the Study and Simulation Cohorts

Characteristic	Study Cohort (*n* = 5631)	Simulation Cohort (*n* = 3000)
Age, mean (SD)	66.7 (9.9)	67.0 (9.2)
Sex (female)	3307 (58.7)	1754 (58.4)
Body Mass Index		
< 24.9 (underweight or normal weight)	632 (11.2)	314 (10.5)
25.0–29.9 (overweight)	1696 (30.1)	912 (30.4)
> 30 (obese)	3305 (58.7)	1774 (59.1)
High Blood Pressure	3020 (53.5)	1613 (53.8)
Back Pain	2074 (36.8)	1080 (36.0)
Diabetes	992 (17.6)	494 (16.5)
Depression	761 (13.5)	322 (10.7)
Heart Disease	663 (11.8)	329 (11.0)

Frequencies and percentages [n (%)] reported for all categorical variables; *SD* Standard Deviation

The patterns of missing data in the study cohort is reported in Table 2. Overall, just 57.4% of the cohort had complete data at both pre- and post-operative occasions. Almost one-third of this cohort had missing data at the post-operative occasion only.

**Table 2:** Missing Data Patterns in the Study Cohort

Pattern	Pre-Operative	Post-Operative	%
1	O	O	57.4
2	O	X	32.3
3	X	O	5.9
4	X	X	4.4

O – Observed; X – Missing

### Regression results for the study cohort

The mixed-effects regression model results for the study cohort on both the SF-12v2 PCS and MCS measures for the intercept, time, and time-sex effects are presented in Table 3. Parameter estimates, standard errors and 95% CI width are provided for the CCA, ML and MI methods. Overall, the three methods did not differ on statistical significance of the parameter estimates for the intercept, time, and time-sex. However, there were differences amongst the methods for the coefficients of age, diabetes, and heart disease in the model for MCS, which were not statistically significant for the CCA method but were statistically significant for the ML and MI methods (estimates not shown). Overall, the CCA method yielded 95% CIs that were substantially wider than for the ML and MI methods. ML and MI produced similar estimates and 95% CI widths (see Table 3).

**Table 3:** Mixed-Effect Regression Model Parameter Estimates for the SF-12v2 PCS and MCS Scores

	Model Effect	CCA		ML		MI	
		Estimate (SE)	CI Width	Estimate (SE)	CI Width	Estimate (SE)	CI Width
PCS	*β*_0_	**37.85 (1.10)**	4.32	**35.91 (0.86)**	3.36	**37.72 (0.92)**	3.65
	*β*_*T*_	**12.42 (0.30)**	1.18	**12.67 (0.27)**	1.04	**12.57 (0.26)**	1.03
	*β*_*TS*_	**−1.03 (0.39)**	1.53	**−1.35 (0.35)**	1.36	**−1.34 (0.35)**	1.39
MCS	*β*_0_	**54.20 (1.32)**	5.18	**50.86 (1.07)**	4.19	**51.15 (1.07)**	4.22
	*β*_*T*_	−0.24 (0.29)	1.13	0.01 (0.27)	1.06	0.004 (0.25)	0.99
	*β*_*TS*_	**1.13 (0.38)**	1.48	**1.34 (0.35)**	1.39	**1.48 (0.33)**	1.32

*CCA* Complete Case Analysis, *ML* Maximum Likelihood, *MI* Multiple Imputation, *HBP* High Blood Pressure, *CI* Width Width of the 95% confidence interval, *SE* Standard Error, Boldface font indicates statistically significant estimates at *α* = 0.05

### Simulation study results

The performance measures for the computer simulation, including the standardized bias, RMSE, and average width of the 95% CI for the CCA, ML, and MI methods are reported in Table 4. The 95% CI coverage (not reported) for the CCA, ML and MI methods ranged between 95% and 97% when data were MCAR, between 91% and 97% when data were MAR and between 60% and 93% when data were MNAR. The lowest number in each of these sets of values corresponds to the case when 50% of the data were missing.

**Table 4:** Simulation Performance Measures for Complete Case Analysis (CCA), Maximum Likelihood (ML) and Multiple Imputation (MI)

Missing Mechanism	Model Parameter	Performance Measure	10% Missing			25% Missing			50% Missing		
			CCA	ML	MI	CCA	ML	MI	CCA	ML	MI
MCAR	*β*_0_	Bias	5.57	6.90	7.53	2.03	1.09	3.97	−0.19	1.51	6.23
		RMSE	1.89	1.83	1.84	2.14	1.96	1.97	2.68	2.19	2.23
		CI Width	8.28	8.09	8.12	9.06	8.48	8.56	11.14	9.31	9.50
	*β*_*T*_	Bias	−2.91	−3.29	−11.63	−3.47	−1.79	−20.61	− 1.90	−2.21	− 39.96
		RMSE	0.47	0.47	0.45	0.54	0.52	0.49	0.68	0.60	0.53
		CI Width	2.18	2.15	2.15	2.38	2.29	2.31	2.92	2.62	2.64
	*β*_*TS*_	Bias	3.21	3.27	15.30	3.30	0.89	29.48	−4.47	−2.62	60.89
		RMSE	0.59	0.59	0.55	0.68	0.66	0.56	0.86	0.76	0.58
		CI Width	2.85	2.81	2.81	3.12	3.00	3.00	3.81	3.43	3.32
MAR	*β*_0_	Bias	14.08	6.19	7.17	11.42	0.56	2.01	−13.98	−9.28	−6.69
		RMSE	1.87	1.81	1.81	2.08	1.91	1.92	2.59	2.12	2.16
		CI Width	8.21	8.05	8.08	8.89	8.39	8.47	10.67	9.12	9.24
	*β*_*T*_	Bias	20.71	14.92	9.90	42.60	32.55	21.65	86.01	64.71	43.61
		RMSE	0.47	0.46	0.44	0.56	0.52	0.47	0.81	0.66	0.52
		CI Width	2.14	2.12	2.13	2.30	2.23	2.25	2.72	2.51	2.56
	*β*_*TS*_	Bias	11.25	10.53	21.15	21.13	18.10	45.06	10.25	3.52	64.01
		RMSE	0.60	0.59	0.56	0.69	0.66	0.59	0.85	0.75	0.59
		CI Width	2.83	2.80	2.81	3.09	2.98	2.98	3.79	3.41	3.31
MNAR	*β*_0_	Bias	−52.67	−41.61	−39.78	− 111.56	− 82.51	−80.01	− 171.53	− 105.67	− 102.79
		RMSE	2.05	1.89	1.89	3.08	2.45	2.43	5.23	3.16	3.17
		CI Width	8.13	7.95	7.97	8.79	8.27	8.34	10.70	9.16	9.35
	*β*_*T*_	Bias	−1.46	7.61	−2.71	−25.40	−9.78	−36.13	−73.72	−47.93	− 110.44
		RMSE	0.47	0.47	0.45	0.57	0.53	0.51	0.87	0.69	0.76
		CI Width	2.19	2.15	2.17	2.42	2.32	2.34	3.04	2.70	2.73
	*β*_*TS*_	Bias	−17.53	−18.53	−5.93	−32.42	−34.40	−3.92	−45.61	−45.24	17.64
		RMSE	0.59	0.59	0.54	0.72	0.69	0.54	0.99	0.88	0.53
		CI Width	2.85	2.81	2.82	3.13	3.01	3.02	3.90	3.50	3.41

*CI Width* Average width of the 95% confidence interval, *Bias* Standardized bias, *RMSE* Root mean squared error

The RMSE and the average width of the 95% CI increased as the rate of missingness increased, reflecting the expected loss of information that occurs with increased rates of missing data. Under the different missing data mechanisms, the average 95% CI width and RMSE obtained from the ML and MI methods were similar for all main effects, but not for the two-way interaction. When 25% to 50% of the data were missing, the average width of the 95% CI from the ML method was marginally narrower than the width for the MI method. The standardized bias for the CCA, ML and MI methods when data were MNAR was twice the size of the bias observed when the data were missing because of MCAR and MAR mechanisms. As the rate of missingness increased, the standardized bias also increased. However, when the missing data were MCAR, the standardized bias for the MI and ML methods were larger than for the CCA method, while the RMSE of the CCA method was substantially larger than for the MI and ML methods.

Simulation results for the MI and MI-Aux methods are reported in Table 5. Including the hypothetical auxiliary variable in the imputation model reduced the average width of the 95% CI as its correlation with the outcome variable increased. The size of the reduction increased as the rate of missing data increased. When 50% of the data was missing, we obtained a reduction of up to 14% and 4% in the average 95% CI width by including the hypothetical auxiliary variable with *ρ* = 0.8 and 0.5, respectively. However, there was no significant reduction in the average 95% CI width when the hypothetical auxiliary variable with *ρ* = 0.2 was included in the model. Similarly, a reduction of up to 5% and 2% in the average 95% CI width was observed when the percentage of missing data was 25% and 10% respectively.

**Table 5:** Simulation Performance Measures for Multiple Imputation (MI) without and with an Auxiliary Variable (MI-Aux)

Missing Mechanism	Model Parameter	Performance Measure	10% Missing				25% Missing				50% Missing			
			MI	MI-Aux			MI	MI-Aux			MI	MI-Aux		
				*ρ* = 0.2	*ρ* = 0.5	*ρ* = 0.8		*ρ* = 0.2	*ρ* = 0.5	*ρ* = 0.8		*ρ* = 0.2	*ρ* = 0.5	*ρ* = 0.8
MCAR	*β*_0_	Bias	4.48	3.75	3.70	3.29	2.31	3.05	3.25	0.74	3.89	4.81	5.05	4.36
		RMSE	1.78	1.78	1.77	1.74	1.88	1.89	1.85	1.77	2.19	2.18	2.11	1.94
		CI Width	8.11	8.09	8.03	7.92	8.58	8.53	8.39	8.09	9.50	9.42	9.10	8.46
	*β*_*T*_	Bias	−2.16	−2.06	−0.54	2.18	−19.72	−21.47	−21.49	−12.81	−36.70	− 37.14	−31.22	−16.90
		RMSE	0.46	0.46	0.46	0.45	0.47	0.46	0.46	0.44	0.52	0.53	0.52	0.47
		CI Width	2.16	2.16	2.15	2.12	2.32	2.31	2.29	2.21	2.65	2.64	2.57	2.38
	*β*_*TS*_	Bias	4.18	3.84	2.61	−1.39	29.38	32.62	33.04	21.74	61.45	62.28	52.82	29.08
		RMSE	0.56	0.56	0.56	0.56	0.57	0.57	0.57	0.56	0.60	0.60	0.60	0.58
		CI Width	2.82	2.82	2.81	2.77	3.00	3.00	2.96	2.88	3.34	3.31	3.25	3.05
MAR	*β*_0_	Bias	−1.29	−0.56	0.67	3.06	−11.52	−10.87	− 10.59	−8.20	−26.62	− 19.67	− 10.71	4.93
		RMSE	1.77	1.76	1.76	1.74	1.87	1.88	1.84	1.76	2.26	2.24	2.13	1.95
		CI Width	8.05	8.04	8.01	7.93	8.44	8.45	8.32	8.10	9.34	9.31	9.03	8.44
	*β*_*T*_	Bias	9.13	6.53	4.63	3.17	−4.55	−11.15	−18.72	−18.78	−14.41	−14.83	− 10.96	−2.48
		RMSE	0.46	0.46	0.46	0.46	0.45	0.46	0.46	0.45	0.52	0.51	0.50	0.47
		CI Width	2.15	2.15	2.14	2.12	2.31	2.30	2.28	2.21	2.62	2.60	2.54	2.35
	*β*_*TS*_	Bias	−8.19	−6.72	−6.81	−5.64	15.21	20.60	24.92	21.05	31.05	30.19	22.37	6.38
		RMSE	0.57	0.57	0.57	0.57	0.54	0.55	0.56	0.56	0.55	0.54	0.55	0.57
		CI Width	2.83	2.82	2.81	2.77	3.02	3.02	2.99	2.89	3.34	3.32	3.25	3.05
MNAR	*β*_0_	Bias	−42.75	−40.05	−31.76	−13.71	−86.54	−84.55	−73.35	− 42.67	−102.96	− 99.42	−89.10	−53.11
		RMSE	1.89	1.87	1.81	1.73	2.49	2.44	2.26	1.90	3.17	3.10	2.80	2.20
		CI Width	7.97	7.97	7.90	7.84	8.33	8.32	8.20	7.97	9.37	9.27	8.95	8.35
	*β*_*T*_	Bias	7.95	6.41	3.46	1.73	−31.14	−34.74	−35.36	−25.04	− 101.73	− 99.73	−80.58	− 41.96
		RMSE	0.45	0.45	0.45	0.45	0.48	0.48	0.48	0.45	0.74	0.73	0.65	0.51
		CI Width	2.17	2.16	2.16	2.12	2.34	2.35	2.31	2.22	2.74	2.71	2.63	2.40
	*β*_*TS*_	Bias	−16.91	−14.34	−8.65	−3.75	−5.30	1.39	11.72	16.71	16.66	18.66	15.27	8.17
		RMSE	0.55	0.55	0.55	0.56	0.53	0.54	0.55	0.55	0.54	0.54	0.55	0.57
		CI Width	2.82	2.82	2.80	2.77	3.01	3.02	2.98	2.88	3.41	3.39	3.30	3.07

*CI Width* Average width of the 95% confidence interval, *Bias* Standardized bias, *RMSE* Root mean squared error

Inclusion of the hypothetical auxiliary variable in the imputation model reduced the bias and RMSE, particularly in cases where the rate of missing data was high and *ρ* = 0.8. When 50% of the data was missing via an MNAR mechanism, the bias reduction was over 50% and RMSE decreased by up to 45%.

## Discussion

This study used a real-world numeric example and computer simulation to compare several methods for missing data when estimating change in PRO scores from a joint replacement clinical registry. In the numeric example, we investigated the effect of missing data methods on the precision of estimates of change in pre- and post-operative PROs. Standard errors were consistently larger for the CCA method when compared with ML and MI methods. The ML and MI methods produced consistent parameter estimates and standard errors. This is because the imputation and analysis models are similar for both methods [11, 20].

The simulation study investigated the potential benefit of using a supplementary variable on the bias and precision of the MI method. The simulation focused on the effects of a single hypothetical auxiliary variable, although in practice there may be more than one auxiliary variable included in the MI model [20]. The impact on bias and precision was substantial when the amount of missing data was large, and when the correlation between the hypothetical auxiliary variable and the outcome of interest was high. When missingness on the outcome of interest was ignorable, inclusion of an auxiliary variable that was strongly associated with our outcome variable added extra information to the imputation model, which is in agreement with the recommendation of the International Society of Arthroplasty Registries on how to deal with missing data in arthroplasty registries [34]. As a result of this inclusion, we obtained a significant reduction in standard errors, and consequently increased the precision of our analysis, which is consistent with previous research [3, 20, 22]. Furthermore, including an auxiliary variable in the imputation model helped moderate the amount of bias and size of the RMSE when missingness was non-ignorable. Thus, our results are comparable to what would be expected under an ignorable missingness mechanism [20].

Clinical registries may include variables that are correlated with missingness and could therefore be included as auxiliary variables in MI models. However, many variables in clinical registries will have the same pattern of missingness as the outcome of interest. This was true for the clinical registry data used in the current study. Thus, in order to adopt a MI approach with auxiliary variables, the researcher should link the registry data to another data source that has complete information on variables thought to be associated with missingness. For example, it may be possible to link clinical registry data with administrative health data containing measures of healthcare use or diagnoses for comorbid health conditions that may be associated with the presence of missing observations [35, 36]. As well, these data may also contain information about physician characteristics, which may also influence missing data for patients captured in clinical registries. For example, some physicians may focus on more or less complex patients; comorbid characteristics, which are likely to be more common in more complex patients, may have a strong impact on patient dropout/loss to follow-up.

This study has a number of strengths. First, we used a combination of computer simulation and a real-world numeric example to examine the effect of the missing data method on estimates of change in PRO scores. The use of simulated data drawn from the study cohort ensures that the complex relationships amongst the covariates and the outcomes are preserved, which facilitates understanding of the impacts of missing data in real-world settings [23]. We examined change in both the SF-12v2 PCS and MCS scores in our numeric example, and PCS only in our simulation study as we expect the performance measures to have similar patterns across outcomes. There are, however, some limitations to this study. In our simulation study, we considered only the hypothetical situation of using a single auxiliary variable in the imputation model due to the substantial computation time for the simulation study. Also, we only considered the case where the relationship between the auxiliary variable and outcome of interest was linear. It is possible to have a scenario where the relationship is non-linear. Moreover, we did not include an auxiliary variable in our numeric example. The linkage of the WRHA Joint Replacement Registry to sources of auxiliary variables, such as administrative health data, requires data access approvals and a health information privacy impact assessment. Moreover, the choice of one or more potential auxiliary measures to include in a MI model is not a straightforward process; both theoretical and practical considerations must be addressed, which is beyond the scope of the current paper [20].

## Conclusion

In summary, we examined the impact of different missing data mechanisms and an auxiliary variable on the bias and precision in estimating change over time in PROs. Our simulation results showed that using auxiliary information in the imputation model can increase the precision and reduce the bias of parameter estimates, especially in cases where the percentage of missing data is high.

In the absence of an auxiliary variable, the simulation results revealed that the ML method is more precise in estimating longitudinal change in PRO measures than the MI method, especially when there is complete data on the covariates. However, MI offers an advantage of straightforward inclusion of one or more auxiliary variables in the imputation model over the ML method. Under the expectation of inevitable missing data when conducting a longitudinal study, complete auxiliary information should be collected, such as other measures of the PRO of interest and/or variables that may be associated with the outcome. Our results showed a consistent pattern in all the scenarios considered. Therefore, we recommend that in the presence of missing data, initial analyses should be conducted assuming MAR and then sensitivity analyses should be conducted assuming MNAR.

## Data Availability

Data used in this article were derived from health data as a secondary source. The data were provided under specific data sharing agreements only for the approved use. The original source data are not owned by the researchers and as such cannot be provided to a public repository. The original data source and approval for use has been noted in the acknowledgments of the article. Where necessary and with appropriate approvals, source data specific to this article or project may be reviewed with the consent of the original data providers, along with the required privacy and ethical review bodies.

## Abbreviations

CCA: Complete case analysis
CI: Confidence interval
MAR: Missing at random
MCAR: Missing completely at random
MCMC: Markov Chain Monte Carlo
MCS: Mental component summary
MI: Multiple imputation
MI-Aux: Multiple imputation with auxiliary variable
ML: Maximum likelihood
MNAR: Missing not at random
PCS: Physical component summary
PRO: Patient reported outcome
RMSE: Root Mean Squared Error
SF-12v2: Short form survey version 2
TKA: Total knee arthroplasty
WRHA: Winnipeg Regional Health Authority
